# Radon concentration in self-bottled mineral spring waters as a possible public health issue

**DOI:** 10.1038/s41598-019-50472-x

**Published:** 2019-10-03

**Authors:** C. Di Carlo, L. Lepore, G. Venoso, M. Ampollini, C. Carpentieri, A. Tannino, E. Ragno, A. Magliano, C. D’Amario, R. Remetti, F. Bochicchio

**Affiliations:** 1 Italian National Institute of Health, National Center for Radiation Protection and Computational Physics, Viale Regina Elena, 299 - 00161 Rome, Italy; 2 Sapienza - University of Rome, Department of Basic and Applied Sciences for Engineering, Via Antonio Scarpa, 14 - 00161 Rome, Italy; 3 National Research Council (CNR), Prevention and Protection Service Office, P.le A. Moro, 7- 00185 Rome, Italy; 4 Ministry of Health, General Directorate for Health Prevention, V.le Giorgio Ribotta, 5 - 00144 Rome, Italy

**Keywords:** Risk factors, Hydrology

## Abstract

Since 2013, the Council Directive 2013/51/Euratom has been regulating the content of radioactive substances in water intended for human consumption. However, mineral waters are exempted from this regulation, including self-bottled springs waters, where higher radon concentration are expected. Therefore, a systematic survey has been conducted on all the 33 mineral spring waters of Lazio (a region of Central Italy) in order to assess if such waters, when self-bottled, may be of concern for public health. Waters have been sampled in two different ways to evaluate the impact of bottling on radon concentration. Water sampling was possible for 20 different spring waters, with 6 samples for each one. The results show that 2 (10%) of measured mineral spring waters returned radon concentrations higher than 100 Bq L^−1^, i.e., the parametric value established by the Council Directive. These results, if confirmed by other surveys involving a higher number of mineral spring waters, would suggest regulating also these waters, especially in countries like Italy for which: (*i*) mineral water consumption is significant; (*ii*) mineral concession owners generally allow the consumers to fill bottles and containers, intended for transport and subsequent consumption, directly from public fountains or from fountains within the plant; (*iii*) the consumers’ habit of drinking self-bottled mineral water is widespread.

## Introduction

Exposure to radon dissolved in drinking waters can derive indirectly from the inhalation of air containing radon degassed from water (as during showers or dishwashing, due to the volatility of dissolved radon gas which increases with temperature) and directly from ingestion of water^1,2^. Due to the relatively low solubility of radon in water, about 90% of the dose attributable to radon in drinking waters comes from inhalation rather than ingestion^3^. Nevertheless, the latter exposure pattern should not be neglected as a potential risk for public health^4^.

The Directive 2013/51/Euratom for the protection of the health of the general public with regard to radioactive substances in water intended for human consumption contains several requirements to Member States about radon concentration in water, including: (i) to adopt a parametric value (equal to 100 Bq L^−1^) above which the risk has to be evaluated and remedial actions have to be considered, and (ii) to carry out representative surveys in order to identify water sources whose radon content might exceed such a parametric value^5^.

However, mineral waters are exempted from radioactivity control by the Council Directive 2013/51/Euratom even if their consumption is significant in some countries. In particular, Italy is the first country in Europe for mineral water consumption, with more than 200 L per person per year^6^. This exemption refers both to bottled and not-bottled mineral waters.

As regards to bottled waters, population exposure to radon concentration in such waters is usually low because radon half-life is much shorter than the typical time needed by bottled waters to reach consumers’ houses. As a consequence, radon concentration measured in mineral bottled waters is usually lower or much lower than its above-mentioned parametric value e.g.^7^.

About non-bottled mineral waters, radon exposure can be not negligible when consumers can fill bottles and containers directly from public fountains or from fountains within mineral spring water plants, thus reducing significantly the time elapsing between mineral water bottling and subsequent consumption. In Italy, few radon concentration measurements were performed in mineral spring waters^8^, whereas several surveys were carried out on radon concentration in groundwaters and tap waters since the 90 s (see ^9^ and references therein). Therefore, a survey addressing radon concentration in all the 20 natural mineral spring waters of Lazio (a region of Central Italy) was planned and conducted in order to assess if such waters, when self-bottled, may be of concern for public health. This region has been chosen because its soil is largely made of materials of volcanic origin, such as tuff and pozzolana, leading potentially to high levels of radon in waters.

## Results and Discussions

The mean radon concentration in water from each source was estimated as the average of the values returned by the measurements of three samples collected one after the other. Two sampling method (i.e., *preventive* and *typical* one, described in detail in section 3.2), have been considered for each mineral water in order to investigate the effect of filling method on radon degassing.

The obtained results, expressed in terms of average ± standard error (equal to the ratio of standard deviation of the 3 measurements results to $\sqrt{3}$) of radon concentration, are shown in Table 1. The results are grouped in the table according to the region division into six sampling areas.

**Table 1:** Radon concentration in all the mineral spring waters analysed, expressed in [Bq L^−1^] and computed as the average of the three samples collected in the so-called “preventive” and “typical” approaches.

Area denomination	Municipality	Measurement ID	*“Preventive” Sampling*	*“Typical” Sampling*
			*C*_Rn_ [BqL^−1^]	*C*_Rn_ [BqL^−1^]
*North-West Basin of Tiber River*	Capranica (VT)	NWT1	253 ± 13	236 ± 22
	Nepi (VT) Sulfur	NWT2	29 ± 2	28 ± 2
	Nepi (VT)	NWT3	35 ± 2	29 ± 1
*North-Est Basin of Tiber River*	Città Reale (RI)	NET1	4 ± 1	4 ± 1
	Rieti (RI)	NET2	5 ± 1	5 ± 1
	Sant’Anatolia (RI)	NET3	4 ± 1	3 ± 1
*Downtown Area of Rome*	Roma (RM)	R1	6 ± 1	5 ± 1
	Roma (RM)	R2	22 ± 2	13 ± 1
	Roma (RM)	R3	5 ± 1	4 ± 1
	Roma (RM)	R4	2 ± 1	2 ± 1
*Aniene River Valley*	Marano Equo (RM)	A1	25 ± 2	27 ± 1
	Marano Equo (RM) Magnesian	A2	55 ± 4	
	Fiuggi (FR)	A3	37 ± 1	
	Guarcino (FR)	A4	3 ± 1	4 ± 1
*Pontino Plain Area*	Rocca Priora (RM)	PP1	38 ± 3	31 ± 2
	Aprilia (LT)	PP2	28 ± 3	20 ± 1
	Aprilia (LT)	PP3	12 ± 1	7 ± 1
	Aprilia (LT)	PP4	256 ± 14	176 ± 11
*Sacco and Liri-Garigliano Rivers Basin*	Gavignano (RM)	SLG1	59 ± 3	64 ± 4
	Minturno (LT)	SLG2	17 ± 2	13 ± 1

The uncertainties are expressed with coverage factor (k) equal to 1.

For mineral spring waters A2 and A3, “typical” results are not available due to the restrictions adopted by the owners about the number of bottles non-residents can fill: in such a condition, the three containers were all filled in the “preventive way”.

**Figure 1:**
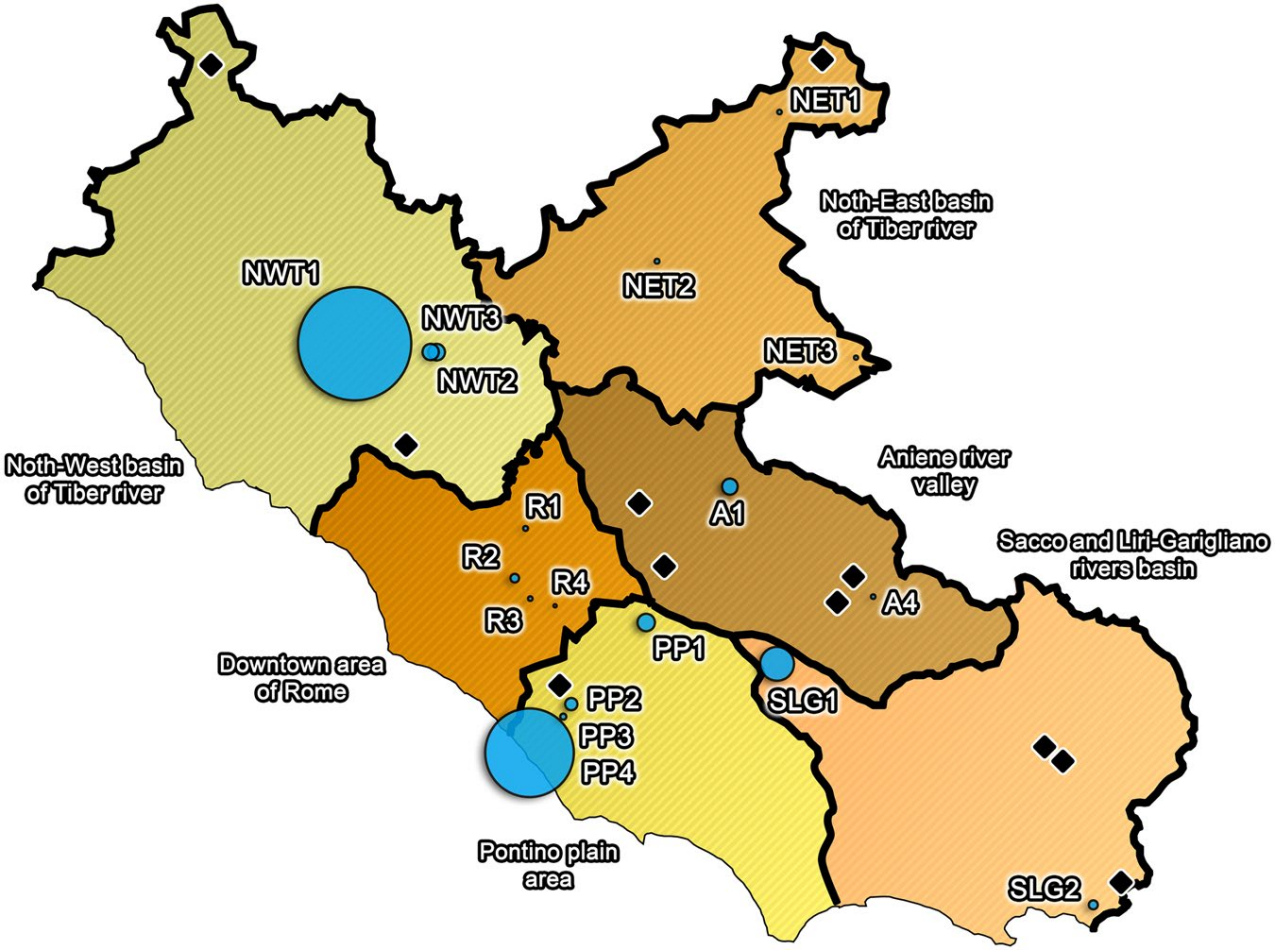
Radon concentration distribution in mineral spring waters of Lazio sampled in the so-called “*typical*” way. Each mineral spring water source is associated to a circle of dimension proportional to radon content measured. Thick lines represent the boundaries dividing the territory into the six sampling areas discussed above. The black diamonds identify the position of the mineral spring water plants whose samples could not be collected during the survey. *The image has been created through GIMP 2*.*10*.*12* (https://www.gimp.org/).

The measurement identification codes, reported in the third column of Table 1, allow associating the proper radon concentration of each source to the corresponding circle of Fig. 1.

**Figure 2:**
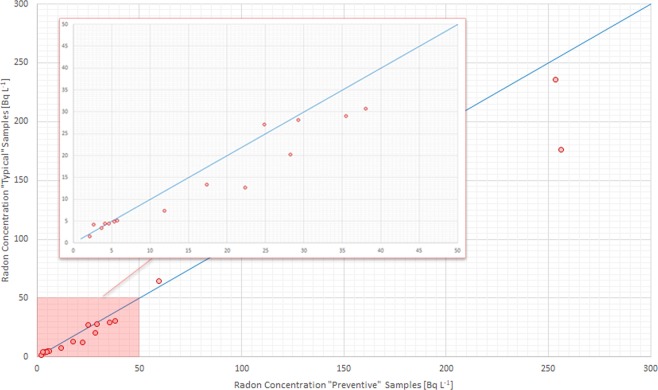
Radon in water concentrations obtained through *typical* versus *preventive* sampling. The blue solid line denotes the equality of the two variables (i.e., *y* = *x*). The dashed line denotes a situation where *y* variable is 30% lower than *x* variable (i.e., *y* = 0.7*x*). The region 0–50 Bq L^−1^ is zoomed in the red box. The uncertainties are expressed with coverage factor (k) equal to 1.

As expected, radon concentration measured from samples obtained in *typical* way is generally lower (for about 80% of the cases) than the corresponding values obtained using the *preventive* sampling method. However, considering the uncertainties, the differences between the two approaches are statistically significant only for 3 cases (about 20% of the total) and the relative difference between to two approaches is mostly lower than 30% (see Fig. 2), also for radon levels higher than 100 Bq L^−1^.

Hence, the *typical* sampling leads to a radon loss not so large as compared to a more careful sampling procedure. Since *typical* sampling reproduces the typical consumer’s handling for the water sampling from the fountains, it can be considered more representative of the actual radon levels than the other sampling approach. Therefore, data analysis and comparison with previous studies will be hereafter carried out using data of radon concentration in water measured with the *typical* sampling approach.

Radon concentration levels measured in the surveyed spring waters follow a right-skewed distribution, similarly to the usual log-normal distribution of indoor radon concentration levels. In fact, generally low levels of radon concentration were found: 50% of them has values lower than 13 Bq L^−1^ and 75% of them has values lower than about 30 Bq L^−1^. The variability of radon concentrations in spring waters within the same area is quite high (Table 1), mostly higher than 80% if expressed in terms of coefficient of variation of radon levels. The high data variability within areas and the presence of mostly low radon levels were also found in a previous study on radon concentration in different types of waters conducted in 14 Italian regions^8^, including Lazio region, although limited to the Province of Viterbo (VT), approximately in the same *North-West Basin of Tiber River* area of the present study. In this area, the authors found a high fraction of tap and spring waters having high radon levels: 25% of them higher than 87 Bq L^−1^ with a maximum of 624 Bq L^−1^ ^8^. These results are similar to those of the present study and can be explained with the fact that soils of Viterbo province are mainly on volcanic origin, with an high concentration of natural radionuclides, including radium which decays into radon^10^.

The present study shows an exceedance of 100 Bq L^−1^, the parametric value for radon in water intended for human consumption established by Council Directive 2013/51/Euratom^5^, for two mineral spring waters in two different areas, one in *North-West basin of Tiber river* and one in *Sacco and Liri-Garigliano rivers basin*. The presence of waters exceeding the parametric value was previously found in only 5 out of 14 Italian regions, including also spring and tap waters of Lazio^8^.

Considering all the samples of the present survey (see Table 1), the percentage of mineral waters exceeding 100 Bq L^−1^ is 10%. This percentage is one of the highest found for spring and mineral water, both in Italy and in some European countries (see Table 2). However, it is worth noting that this percentage was calculated on a relatively low numbers (20) of mineral water samples and further studies are needed to evaluate if similar data are present also in other Regions.

**Table 2:** Percentages of water samples exceeding 100 Bq L^−1^, the parametric value for radon in water according to the Council Directive 2013/51/Euratom, estimated in different types of waters and areas.

Area	Type of waters	N of samples	Exceedances of 100 Bq L^−1^[%]	Reference Paper
***Italian region***				
Umbria	Natural spring waters	18	0%	^22^
Lazio	Mineral spring waters	20	10%	Present study
Trento province	Thermal and mineral waters	20	0%	”
Veneto	”	37	8%^a^	”
Piemonte and Valle d’Aosta	”	57	0%	”
Liguria	”	37	0%	”
Sicilia	”	12	0%	”
Sardegna	”	30	79%^a^	”
***Other European Countries***				
South of Catalonia (Spain)	Natural spring waters	15	7%	^23^
Balaton Highland and South Hungary (Hungary)	”	44	7%	^24^
Bulgaria	Mineral waters	54	28%	^25^
Romania	Natural spring waters	137	<1%	^26^

Only surveys with more than 10 samples of waters are reported.

^a^These exceedances were estimated from data of Table 2 in Giovani *et al*.^8^, in which average and median of data for each survey are reported, assuming a lognormal distribution of the data and putting the geometric mean equal to the median.

## Materials and Methods

### Surveyed mineral spring waters

In Lazio, a total of 33 mineral spring water concession (MSWCs) are present until 2018, according to the number of authorizations granted by the regional authority to public and private subjects^11^. These concessions are distributed in six areas of the region (showed in Fig. 1). The sampling operations interested only the 18 MSWCs of Lazio whose concession owner allows people to self-bottle directly from municipal public fountains or from fountains within the plants for industrial bottling. However, two of the sampled MSWCs actually manage two different spring waters, so that the total number of different waters included in this survey rises to 20 (Table 3).

**Table 3:** Summary of mineral spring water concessions (MSWCs) involved in the present survey and different waters sampled.

Area of Lazio	Total number of MSWCs	Number of sampled MSWCs	Number of different waters sampled
*N-W basin of Tiber river*	6	2	3^a^
*N-E basin of Tiber river*	4	3	3
*Downtown area of Rome*	4	4	4
*Aniene river valley*	7	3	4^a^
*Sacco and Liri-Garigliano rivers basin*	6	2	2
*Pontino plain area*	6	4	4
*Total*	33	18	20

Two plants, one in the municipality of Nepi (VT) and one in the municipality of Marano Equo (RM), manage two different waters with specific physical-chemical properties.

For some concessions (13), measurements could not be performed due to different reasons. Some plants (6) were closed/inoperative (or actually did not exist) during the survey. One plant was seriously damaged by a recent earthquake. In two plants, two different concessions were managed and it was not possible to distinguish them. For two concessions (both distributed via public fountains), the fountains supplied by the source were not identified. In all other cases (4), the policy adopted by the concession companies does not allow the consumer to fill bottles or containers directly at the source within the plant.

### Water sampling procedure

The radon concentration is evaluated at the point where the water is put into containers for two main reasons:

i. The radon concentration in samples collected directly at the plant is higher than in any other scenario interesting the same source. Indeed, referring to bottled water, the radon concentration in water stored in containers for transport and subsequent consumption decreases due to the natural radioactive decay of Rn and the leakage through the sealing of the bottles. Besides, the operations of packing and bottling could highly influence the radon concentration in water, reducing it.
ii. Sampling the water inside the plant allows to know and minimize the time elapsing between the collection and the first opening of the bottle.

Water was collected in polyethylene terephthalate (PET) bottles. It was observed that polyethylene terephthalate (PET) has generally lower radon loss during storage^12,13^ than high density and low-density polyethylene^14^. The material and the sealing are compliant with ISO 13164–1:2013^15^ and ISO 13164-3:2013^16^ concerning principles to be adopted in water sampling, storage, and transportation. All the containers, having a volume larger than 1L, were filled to the brim and plugged such to avoid air volumes between the free surface and the cap. Polyethylene terephthalate was chosen in order to have the possibility of squeezing the bottle when capping, such reducing the air gap and radon diffusion towards the gas phase, too.

For each spring, three samplings were carried out in the so-called *preventive way*. Such filling method aims to obtain a near laminar water flux that avoids spontaneous degassing of dissolved gases when filling the bottle. *Preventive way* was obtained by inclining the bottle and reducing the water flow rate at the minimum value. The remaining three samples were collected in *typical way*, with a medium water flux and by simply placing the bottle in vertical position during filling operation, as a common user would have done.

The sealed samples were then transported to the Italian National Institute of Health where the radon concentration measurements were performed. The time delay between the sample collection and measurements was always kept below 24 h in order to increase measurements precision and to reduce radon loss due to diffusion through PET.

### Measurement system and procedure

The measurements were carried out by means of three AlphaGUARD PQ2000 PRO (Bertin Instruments®) to measure radon concentration, and three AquaKIT (Bertin Instruments®) accessory for samples degassing. According to^17^, the emanometry techniques relying on ionization chamber are characterized by a low detection limit (0.3 Bq L^−1^) and a typical uncertainty (coverage factor *k* = 1) ranging between 5% and 12%.

**Figure 3:**
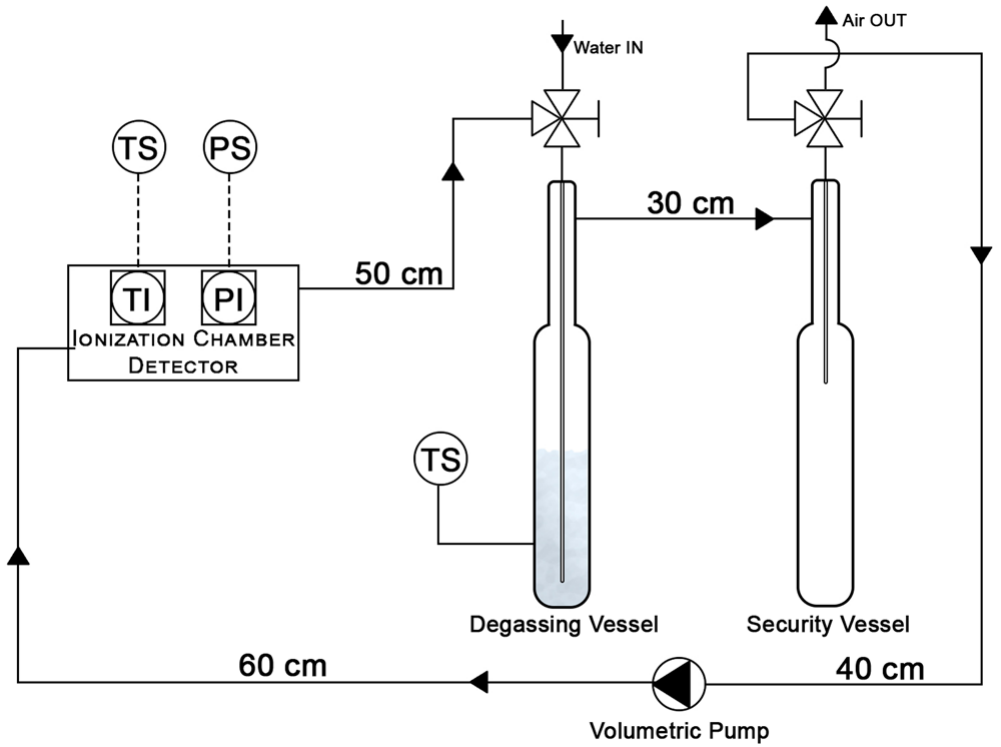
Schematic representation of radon in water concentration experimental setup. The diagram shows the position where temperature and pressure are monitored. Attention should be paid to tubes length and internal diameter when computing the inner volume of the whole apparatus.

The measuring set-up (Fig. 3), consists of: (*i*) a degassing vessel, a custom gas washing vessel of DURAN® that hosts the degassing process; (*ii*) a security vessel, a DURAN® container to collect all the water drops in the gas flow; (*iii*) an active coal filter, used to reduce the radon content in the measurement set-up before injecting the sample; (i*v*) an Alpha Pump (Bertin Instrumens®); (*v*) six connecting tubes, Tygon® connections of different length and with an interior diameter of 4 mm (5/32″).

It is important to care that:

- the lower nozzle of the degassing vessel is connected to the lower nozzle of the security vessel;
- the upper nozzle of security vessel is connected to the volumetric pump inlet;
- the volumetric pump outlet is connected to the inlet of ionization chamber;
- the ionization chamber outlet is connected with the upper nozzle of the degassing vessel such to close the circuit.

When all previous requirements are satisfied, the pressure head by the volumetric pump overcomes the hydraulic head of the circuit preventing the water from flowing backward the ionizing chamber of the continuous radon monitor.

The radon concentration in water results from the following equation:

1 $${C}_{water}=\frac{{C}_{air}\cdot (\frac{{V}_{system}-{V}_{sample}}{{V}_{sample}}+K)-{C}_{0}\cdot (\frac{{V}_{system}-{V}_{sample}}{{V}_{sample}})}{1000}$$

where:

- *C*_*water*_ is the radon concentration in the water sample [Bq L^−1^];
- *C*_*air*_ is the radon concentration [Bq m^−3^] of the air flowing in the measuring system during the degassing process of water samples. The radon concentration is monitored by the detector, whose functioning mode is set to 1 min FLOW, for 20 minutes. The air flow rate is set to 0.5 L min^−1^;
- *C*_0_ is the radon concentration [Bq m^−3^] of the air contained in the measuring system before the injection of the sample inside the degassing vessel. The radon concentration is monitored by the detector, whose functioning mode is set to 1 min FLOW, for 10 minutes. The air flow rate is set to 0.5Lmin^−1^;
- *V*_*system*_ is the total volume [mL] of the complete measuring system, 1102 mL ± 1%, according to AquaKIT manual (Genitron Instrument GmbH, 2012; Saphymo GmbH, 2017);
- *V*_*sample*_ is the water sample volume [mL]. All the measurements referred in this paper were performed with a sample volume of 100 mL;
- *K* is the Ostwald absorption coefficient which describes the ratio of the radon concentration in water to the radon concentration in air, at thermodynamic equilibrium. This coefficient has been computed using the following mathematical formula: *K* = 0.105 + 0.405*e*^−0.0502⋅*T*[°C]^^18,19^.

The three measurement chains were used to participate to *REM 2018 Radon-in-water proficiency test* organized by the European Commission Joint Research Centre (JRC) in November 2018. The results quality was evaluated by computing percentage difference D% from the reference value, *z* score and *ζ* score according to ISO 13528:2015^20^. Considering the three measurement chains, D% ranges between 3.1 and 5, *z* score between 0.21 and 0.33, and *ζ* score between 0.37 and 0.59, being the “satisfactory levels” 15%, 2, and 2 respectively^21^. The measurement protocol will be the subject of a further paper concerning the repeatability and reproducibility of measurements performed by three identical measurements chains.

## Conclusions

A systematic survey on radon concentration in natural mineral spring waters located in Lazio (Central Italy) were carried out in order to evaluate if radon levels in mineral spring waters, exempted by the Council Directive 2013/51/Euratom^5^, may be of public health concern as well as other types of waters intended for human consumption. The sampling protocols allowed to measure radon concentration in mineral spring waters self-bottled by consumers directly from public fountains or from fountains within industrial plants, which is a habit quite common in several Italian Regions.

Overall, the percentage of the sampled mineral waters exceeding the parametric level of 100 Bq L^−1^, introduced by the Council Directive, resulted to be equal to 10%, although this value is quite uncertain being based on only 2 (out of 20) measured waters. Anyway, this percentage is one of the highest among those found in other surveys addressing natural spring waters (mineral, non-mineral, and thermal).

If the results of this survey will be confirmed by further studies in other Italian Regions and in other countries, requirements regarding radon control in mineral waters should probably be considered for the future legislation, especially for countries where consumption of such waters cannot be considered negligible.
